# The Hospital. Nursing Section

**Published:** 1903-08-01

**Authors:** 


### The Hospital.

### Nursing Section.

Contributions for this Section of "The Hospital" should be addressed to the Editor, "The Hospital" Nursing Section, 28 & 29 Southampton Street, Strand, London, W.C.

No. 879.—Vol. XXXIV. *SATURDAY, AUGUST* 1, 1903.

### Notes on News from the Nursing World.

### THE QUEEN AND THE JUBILEE NURSES.

The presentation by the Queen of badges and brassards to a number of Jubilee nurses who completed training in the Dublin homes since January 1st, 1903, was the first function undertaken by her Majesty in Dublin last week. The ceremony took place in St. Patrick's Hall, and Lord Gosford, having first presented to the Queen Miss Lamont, Superintendent of the Jubilee Institute in Ireland, her Majesty handed that lady the gold badge of the Institute. Badges on completion of training were then given by her Majesty to Nurses M. Finn, A. Keogh, E. M'Coy, E. Moore, E. Tate, and L. Trinham ; and a certificate for two years' service to Nurse E. M'Killen. The Queen also received Nurses Cusack, Wills and Farrelly, who start work immediately in the West of Ireland, under the Countess of Dudley's scheme, to which her Majesty has subscribed the sum of £50. Miss F. F. Howell, Superintendent of St. Patrick's Nurses' Home, and Miss Horan, Superintendent of St. Lawrence's Training Home, were among the members of the Council of the Jubilee Institute present at the ceremony. When the Queen was handing badges to the nurses who are going to work in the West of Ireland, she said, "God bless you, nurse, I hope you will be happy in your work in the west." No other words were spoken, but the sweet smile which accompanied each presentation will be treasured in the memories of the recipients.

### THE ADDRESS TO THE KING AND QUEEN.

Among the many addresses presented to the King and Queen in Dublin was one from the matrons and nurses of the city. The wording is as follows:—"To their Most Excellent Majesties King Edward VII. and Queen Alexandra—May it please your Majesties—We, the Matrons and Nurses of Dublin, desire to offer a respectful welcome to our gracious King and Queen. We are deeply sensible of the great consideration which your Majesties have always manifested for the welfare of nurses, and we are fully conscious of the beneficial impetus given to our work by the continued and munificent interest of your Majesties. We also desire to express our heartfelt gratitude in that your Majesty has been graciously pleased to take the place of our late beloved and revered Queen in all that concerns nurses, and especially in those who undertake the care of the sick poor throughout the Empire. We tender to your Majesties, whom we rejoice to see, our warm gratitude, and we are, your Majesties' faithful and dutiful subjects." The presentation was made by Mrs. Kildare Treacy, Superintendent of the City of Dublin Nursing Institution, and Miss MacDonnell, superintendent of the Richmond Hospital. The address, which was illuminated by Miss Mary E. Simpson, of Miss Croker's School for the Employment of Women, from entirely Celtic designs from the Book of Kells, bears the signatures of the matrons on their own behalf, and those of about 800 nurses in the city. The case containing it is a dainty piece of work, of white poplin, embroidered with violets and shamrocks, with the Imperial crown and Royal cyphers worked in gold embroidery, and it is lined with pale heliotrope poplin, while at the corners, right and left, holding the address in place, are an Irish harp and wreath of shamrocks.

### THE QUEEN AT THE HOSPICE FOR THE DYING.

The Queen, on her arrival on Friday at the Hospice for the Dying at Harold's Cross, Dublin, was conducted over the wards by the Countess of Dudley, Archbishop Walsh, and the Superioress, Mother Gertrude Chamberlaine, having previously received, with a few gracious words of acknowledgment, a handsome bouquet from a little girl named Elsie Pugh, one of the inmates. Both in St. Mary's and St. Joseph's wards, which are set apart for women, her Majesty spoke to every patient and presented a bunch of flowers to each. In many cases her kind words and sympathetic smile brought tears of gratitude into the eyes of the poor sufferers, old and young, who made pathetic efforts to demonstrate their thankfulness. When the Queen left St. Joseph's ward she was conducted to the reception-room, where she signed in the visitors' book the signature "Alexandra," followed by the date. It had been at first arranged that she should then leave the hospital, but it being made known to the Queen that the men in St. Patrick's ward greatly wished to see her, she signified her desire to comply with their request, and here, too, she addressed each patient and gave each a bunch of flowers. The Superioress having mentioned that two of the inmates dying slowly of an incurable disease were soldiers—one in the Durham Light Infantry and the other in the Royal Fusiliers—who had seen service in South Africa, her Majesty conversed for a short time with both of them, to their own great delight and the intense satisfaction of the sisters who form the nursing staff.

### THE QUEEN AND THE INCURABLES.

During her visit to Dublin the Queen, on Friday, gave evidence of her practical interest in hospital work by a visit to the Royal Hospital for Incurables at Donnybrook. On her arrival she was received by the lady superintendent, Miss Bradshaw, and several members of the committee, and was conducted through two of the wards by the visiting physician, Dr. Chapman. The main corridor was lined from the entrance door to the first staircase by the nurses of the institution. Her Majesty first visited the female consumptive ward, a large ward containing 26 beds. It is bright and airy, with a good through draught, the woodwork being stained light oak. At the end is an iron staircase outside the building, leading direct to the grounds, and furnishing an easy exit for patients who are able to be out, as well as means of speedy escape in case of fire. The Queen chatted with all the patients, to whom she gave flowers, and before leaving she congratulated the nurses on the appearance of the ward, saying that it was "beautiful." She next visited the large ward on the ground floor, known as the "Marlborough," where she again conversed with the patients, females suffering from various diseases, such as rheumatism and paralysis. Having signed the visitors' book the Queen left the hospital, receiving a hearty cheer from the patients who were in the grounds as she drove off.

### THE KING AND QUEEN AT THE VICTORIA HOSPITAL, BELFAST.

One of the great events of the Royal visit to Belfast on Monday was the opening by their Majesties of the Royal Victoria Hospital, and perhaps the most interesting ceremony from the nurses' point of view, was performed by the Queen, who named the Clarence Ward. Standing in the centre of the ward, and looking up at the beautiful tablet on which are inscribed these words: "Their Majesties King Edward and Queen Alexandra, on the occasion of the opening of this hospital, were graciously pleased to name this ward the 'Clarence,' in memory of their beloved son, the late Duke of Clarence, who is held in affectionate remembrance by the citizens of Belfast," the Queen said, "I name this ward the Clarence in loving remembrance of our son." At the express desire of the King and Queen, Miss Bostock, the matron of the hospital, was presented to them.

### THE QUEEN'S INSPECTION AT LONDONDERRY.

On Tuesday after the King and Queen had visited the City and County Infirmary at Londonderry, where the King laid a memorial stone of the new building, the Queen inspected a number of his Majesty's nurses who had been assembled in Brooke Park. The following were presented to the Queen by the Duchess of Abercorn:—Nurses Higginson, Dodwell, Elliott, Forster, Hamilton, and Scott. Her Majesty, who shook hands with each nurse, also presented to Miss Forster the certificate of Queen Victoria's Jubilee Institute for Nurses.

### NEW QUARTERS FOR THE QUEEN'S JUBILEE INSTITUTE.

The offices of Queen Victoria's Jubilee Institute for Nurses have, we are officially informed, been removed from St. Katharine's Precincts, Gloucester Gate, Regent's Park, to 120 Victoria Street, S.W., to which address all communications should in future be sent. It will be remembered that in May last the Queen presented the Institute with a cheque for £1,000 for the purpose of enabling the governing body to secure more centrally situated headquarters. The advantages of the removal from Regent's Park to Victoria Street are obvious.

### THE ANGLO-AMERICAN NURSING HOME AT ROME.

It is quite certain that King Edward would not last month have paid the Anglo-American Nursing Home in Rome the compliment of sending the Committee a signed portrait print of himself as a mark of sympathy with the work done by the institution on behalf of British patients since its opening, unless he believed the management to be perfectly satisfactory. We, therefore, decline on the mere assurance of the anonymous correspondent of one of the daily papers, to accept the statement, "that a remonstrance as to the treatment which they had received while in the home was sent in by the whole body of nurses." It is, however, important to know on what grounds, if any, this allegation, and the more serious one that the managing committee now refused to deal with the complaints of the nurses, have been made, and we are consequently instituting an inquiry into the matter.

### FOUR YEARS TRAINING AT BATH.

In the interesting statements of the matron of the Royal United Hospital at Bath, which are reported this week in an interview with our Commissioner, Miss Polden deals with the question of the period of training. The board have lately extended the term to four years for probationers who do not pay a premium, the fourth year to be spent either in the hospital as a staff nurse, or on the private nursing staff of the institution. The certificate of competency is still to be given at the end of three years, but it is to be further endorsed at the end of the fourth year. During the fourth year the salary is "not less than £15," and it may be increased at the discretion of the board. We think that although the matron finds no difficulty in obtaining candidates for vacant probationerships £15 is an inadequate salary for a capable nurse in her fourth year. When the matron was appointed in 1900 the training at the Royal United Hospital was for two years in the hospital itself, the nurse joining the private staff the third year. This, we agree with the matron, was not a wise plan, but if, in the interests of the hospital, it is considered desirable to bind probationers for four years, there should be an obvious inducement to them, in the shape of salary, to assent to the conditions.

### THE WORKHOUSE MATRON AS NURSE.

There is a marked contrast between the criticisms which Mrs. Richmond, matron of Luton Workhouse, makes with reference to the report of the Departmental Committee and those of Mr. Grayson, to whom we referred last week. Instead of hoping with the master of the Ipswich Workhouse that nothing will come of the report, Mrs. Richmond not only defends the proposed creation of the qualified nurse, but is generally in favour of the conclusions of the committee. She has also an abounding faith in the judgment of the President of the Local Government Board, to whom she is sure that workhouse matrons may safely leave the status of their position. On this point we think that there are good grounds for the confidence of Mrs. Richmond. Thus, the Southam Board of Guardians lately applied to the Local Government Board for sanction to the matron also acting as nurse, and the latter have informed the Guardians that, providing they appoint someone to generally assist the matron in her duties, they have no objection. The explanation is, of course, that the matron in this case is a trained nurse; and we not only believe, but anticipate that Mr. Long and his department will invariably allow the workhouse matron who is both a trained nurse and a capable administrator to take charge of the nursing in the smaller infirmaries which are not separated from the workhouse.

### THE MIDWIVES BOARD AND THE RULES.

A meeting of the Central Midwives Board was held at the Privy Council Office, Whitehall, S.W., on Thursday last week. The secretary reported that, subject to the confirmation of the board, he had made an arrangement with Messrs. Spottiswoode and Co. for the sale of the board's publications, including the rules, forms, and midwives' roll. The action of the secretary was approved. The secretary was instructed to advertise for a lady clerk at 30s. per week, to enter upon her duties when the board take possession of their new office at 6 Suffolk Street, about the middle of August. A letter was read from the clerk of the council enclosing a copy of the rules as revised by the Privy Council after consideration of the representations made by the General Medical Council, the Home Office, and the Local Government Board. After discussion of the suggested amendments, some of which were accepted and others modified, the secretary was directed to return the rules to the Privy Council, with the observations of the board on the alterations made. It is not anticipated that any difficulty will now arise to prevent the approval of the rules by the Privy Council at an early date.

### GLASGOW NURSES AND THE RAILWAY ACCIDENT.

The terrible railway accident at St. Enoch's Station, Glasgow, took place on Monday at an hour when all the nurses of the Royal Infirmary were on duty, and immediately the sad news was known, a band of willing workers hastened to minister to the needs of the injured passengers. Many of the night nurses volunteered their help, and in a very short time the patients were made as comfortable as possible. It is quite an ordinary event for the infirmary to receive a large number of accident cases, and on Monday they were distributed over a number of wards, so that there might not be too great a strain in any single ward. Many of the nurses are away on holiday leave at present, and it speaks exceedingly well for them that messages were received from one and another offering to return at once if they could be of use.

### DISTRICT NURSING AT SWANSEA.

The meeting of the British Medical Association in Swansea this week supplies a reason for the contention that a list of little more than two hundred subscribers to the Swansea Nursing Association is not creditable to the town. If in size and importance, it is considered worthy of being chosen as the headquarters of the medical profession at their annual gathering, it ought to provide more adequately for the needs of its sick poor. In the circular just issued by the committee of the Nursing Association it is shown that the work is seriously hampered for want of funds. There are at present three nurses on the staff and a fourth is urgently required. But unless the organisation is much better supported the committee will have to reduce, rather than to augment, the number. It is pointed out that if each family in Swansea would subscribe only a shilling a year, all anxiety as to income would be at an end. In pleading for a large body of small subscribers, the committee are going the right way to work, and the Swansea people should mark the event of the year by a response which will ensure the immediate engagement of a fourth nurse.

### NURSES IN THE WILL DISPUTE.

The case of Westoby and another *v.* Grant and another, in which two maiden ladies named Westoby ask for the will of Mrs. Kate Powell, leaving the bulk of her property to Dr. Grant and her solicitor Mr. Griffiths instead of to them, be set aside on the ground of want of testamentary capacity on the part of the testatrix when the will was executed, is of interest, because a trained nurse was a principal witness on each side. Miss Maud Deyns, who gave evidence in favour of the defendants, was the day nurse in attendance on the patient. She could only say that when she saw Mrs. Powell on February 5th, 1903, the date the will in dispute was signed, she "thought" that she understood questions put to her. The other nurse, Miss Florence Nystroom, who was engaged as night nurse, described her experiences in the early morning of February 5th. Between six and seven she was asked to place a pencil in the hand of her patient, and subsequently to guide her hand to make two crosses. She stated in cross-examination that she supposed she was witnessing a will made out by the lady when she was in good health, whereas it appears that she was helping her to sign a document which had been drawn up during that night or in the early morning. We have nothing to do with the main question at issue, but there is a moral to the case so far as nurses are concerned. In their own interest they should absolutely decline to sign any document which is submitted to them for that purpose while they are in attendance upon a patient, unless the opportunity is afforded to them of understanding its nature; and when it comes to guiding the hand of a person who has to be propped up by pillows, they cannot be too particular in insisting upon being fully informed of all details before they are privy to the transaction.

### ROYAL MINERAL WATER HOSPITAL.

We understand that Miss E. Hellings has resigned her appointment as matron of the Royal Mineral Water Hospital, Bath, after upwards of nineteen years' service. She is succeeded by Miss Griffiths.

### NEW NURSES' HOME AT BIRKENHEAD.

Last week the Nurses' Home in Park Road South, Birkenhead, a spacious building admirably suited for the purpose, was opened by the Marchioness of Hertford, mother of the late Lady Margaret Ismay, whose interest in the work of the district nurses was so much appreciated. The entire cost of furnishing the sitting-room was defrayed by Mr. James Ismay, who himself selected the furniture, which is not only comfortable, but in some respects luxurious. In the main, however, comfort rather than luxury has been aimed at by the committee who justly consider it to be their duty to observe a wise economy in the administration of the funds at their command.

### The Nursing Outlook.

"From magnanimity, all fear above;
From nobler recompense, above applause,
Which owes to man's short outlook all its charm."

### THE DISTRICT NURSE.

"Of all the forms that charity takes there is hardly one that is so directly successful as district nursing. It is almost true to say that wherever a nurse enters the standard of health is raised." These are noble words and, what is more to the purpose, they are literal truth. Uttered by a public man of repute they must carry immense weight with all classes, but when we state that they will be found on page 157 of the final volume of "Life and Labour in London," by Mr. Charles Booth (Macmillan), they should convince the most sceptical.

Mr. Charles Booth, whose life work has been the accurate study of the life and labour of the people in London as the metropolis of the Empire, is necessarily a keen observer. He speaks out of fulness of knowledge, and as a practical man tersely but wisely states the conclusions he has arrived at in conjunction with his wife, to whose constant sympathy, help, and criticism he attributes the successful accomplishment of his great task. Like Florence Nightingale, he looks "upon the district nurse, if she is what she should be, and if we give her the training she should have, as the great civiliser of the poor, training them as well as nursing them out of ill-health into good health (health missioners), out of drink into self-control, but all without preaching, without patronising—as friends in sympathy. But let them hold the standard high as nurses." This civilising influence is now at work in all parts of London, and, thanks to Queen Victoria and the Jubilee Nurses' Institute, it has extended to all parts of the United Kingdom. The country little realises the debt it owes to the late Duke of Westminster, the late Mr. William Rathbone, M.P., and many others whose works do follow them.

Much controversy has raged around the question of the amount of training which should be given in nursing to a district nurse before she is allowed to enter upon her vocation. As the result of close study and observation Mr. Charles Booth rejoices that the amount of training and aptitude brought to the work varies very much, for in the work to be done there is room for all grades. "Even if it were possible that all who undertake to help the sick in their homes should be highly trained hospital nurses, this would not be desirable." It is essential that the work of a certificated and trained nurse should be confined strictly to its professional side. Hence the need for such services must constitute the paramount claim. But there are many other duties of the district nurse such as those included in Miss Nightingale's words, which must be undertaken, if district nursing is to exercise the civilising influence which makes it so powerful an agent for good amongst the people. Hence Mr. Charles Booth advocates the employment of parish or mission nurses, for whom a lower standard of training may be accepted, whose duties, already mentioned, "may be regarded in the eyes of those who employ her, or even in her own, as of the very first importance, and as the end to which everything done should conduce." For economical reasons, too, it is essential to encourage and provide the latter class of nurses.

Mr. Booth thus contemplates two systems, one worked entirely by the higher grade nurses possessing great professional skill, and the other by the parish or mission nurses for whom a lower standard of training is accepted. The first class should be collected at one centre, their course of work arranged and supervised by a lady superintendent who is herself a trained nurse, "for so they can accomplish far more, and do it far better, than if distributed amongst the parishes and placed, each individually, under parish control. Their work, however, must be confined strictly to its professional side." Beyond this there is room for "partially skilled women employed locally, in connection with religious or charitable work, to whatever extent they can be provided. There need be no clashing, since in each particular district it is for the doctor to decide as to what degree of nursing skill is essential; and co-operation may even be possible between the more professional and the less professional, especially in cases when the home care is very deficient. As doctor leans on nurse, so might the nurse, who could come in only once in the day, lean on the assistance of her who lived near, and each might learn something from the other." Such are Mr. Booth's proposals, and they are worthy of careful consideration.

Nursing, freed from those who enter its ranks for merely selfish ends, cleansed of everything which tends to bring the name of nurse down from the high atmosphere in which she is placed by Mr. Charles Booth's ideal, attracting only the women who are most womanly, and who regard their calling as a sacred trust, must properly attain to the highest position amongst professions open to women. If it were possible to open windows of understanding and realisation in the minds of the best women amongst all classes, we should find that some of the wisest and best amongst them would deliberately and of set purpose determine to devote their lives to district nursing, because it would afford them an opening where they could contribute inestimably to the upbuilding of the people, the formation of their character, and the realisation of the true nature of happiness.

Apart altogether from religious propaganda, or the inculcation of thrift, or the administration of charity, the district nurse, resident amongst her patients, is often a centre of illumination, and her influence for good through loving service, by the household assistance she renders and the example she sets, confers benefit of inestimable value to the community. The whole population benefits by her services. Her direct influence on the health is considerable, but the gradual educational influence exerted on the common ideas of the people by her teaching as to the special requirements of a sick room, and on such subjects as cleanliness, the care of young children, the preparation of food not for invalids alone, and on many points of minor domestic economy, make her daily life full of absorbing interest to herself and of benefit to her people. Hers is a truly noble mission to raise the standard of life in every house where her loving services are rendered.

### Lectures on Medical and Surgical Nursing.

By John Hopkins, F.R.C.S., Medical Superintendent of the Central London Sick Asylum District Asylums, Cleveland Street, W., and Hendon, N.W.

BLECTURE XIII.—DISEASES OF THE DIGESTIVE SYSTEM.—(*Continued*).

*Obstruction of the Bowel* may be acute or chronic. When acute there is immediate danger to life, and quick relief is imperative.

Obstruction is caused in many ways. The bowel may be invaginated, that is, it may have a tuck in it, and this tuck may grow deeper and deeper until several feet of intestine are turned in. If in turning a stocking you stop half way and putting your hand in at the lower end you pull back the toe till it becomes visible, you have a sample of invagination. The bowel thus affected may descend and protrude from the anus. It sometimes is obstructed, but often motion can pass. The part invaginated becomes strangulated, that is, the circulation is obstructed and inflammation and gangrene results. The part of the gut that is the subject of strangulation is sloughed off and comes away. But the life of the sufferer is in danger and it is necessary to do what is possible to restore the bowel to its normal state before the strangulation has caused the part to slough.

The condition causes paroxysmal pain in the abdomen and vomiting. Relief is sought by injections of air or water into the bowel in order by distending it to release the infolded part. If this fails an operation under an anæsthetic is necessary, when the bowel is taken hold of and the invaginated part pulled out.

*Strangulation* is generally due to compression of the gut either in the neck of a hernial sack or internally by a band across the bowel at some part or a twist in it. There may also be an internal hernia, that is, a knuckle of gut may have slipped into a ring somewhere within the abdomen and this may become strangulated. When there is obstruction, the contents of the bowel cannot pass, and vomiting results. When there is also strangulation there is paroxysmal pain as well. After a while, the contents of the small intestine regurgitate into the stomach and are vomited. There is then a fæcal odour about this vomited matter. If unrelieved, inflammation, gangrene, and peritonitis follow and death results. A strangulated bowel in a hernia can often be returned by the taxis, that is, by manipulating the sack. The taxis is facilitated by a warm bath, in which the taxis may be practised. Failing by this means to reduce the rupture, an operation becomes necessary, for which the part must be prepared by shaving, washing, and the application of an antiseptic pad.

To obviate the danger of strangulation, a hernia may be radically cured. By stitching up the aperture in the abdominal wall through which the rupture protrudes, the subject of the hernia is permanently relieved of all trouble. A truss should always be worn for a hernia when the patient is not in bed. In infants an umbilical hernia is common, but by the constant wearing of a pad, the umbilical opening will often close; but the pad must be kept constantly applied and on no account should the rupture be allowed to come down.

*Chronic Obstruction* leads to a great distension of the abdomen, and active peristaltic movements of the bowel can be seen through the abdominal walls. The seat of obstruction is generally in the large bowel and may be due to simple stricture; but common causes are cancer or pressure on the bowel by tumours. As the obstruction comes on slowly, the action of the bowel is increasingly interfered with, and in order to help the patient both aperients and enemata may be necessary. When stricture, whether cancerous or simple, causes total obstruction, the only relief to be obtained sometimes is by opening the bowel in the side by the operation of colotomy.

*Intestinal Worms* are principally of three kinds: the tapeworm, the round worm, and the thread worm. The tapeworm has a small head, about the size of that of a pin, and a long body divided into segments. Those near the head are small and broader than they are long. As they get farther from the head they get larger, and at the same time increase in length more than in breadth, so that at about three feet distant they are square. Thence they become narrower, but much longer. In treating for this disease, the object is to destroy and cause the head to pass from the bowel, for if this be left behind the worm will grow again. Round worms are like large earth worms in shape and are of a white colour. They may crawl into the stomach and œsophagus and thus come upwards.

In order to clear the intestine of tape or round worms the bowel should be well cleared and no food given till the medicine has had time to destroy them, and then a purgative is necessary to clear them out.

Thread worms are very small and look like bits of thread; they are four-tenths of an inch long only, and inhabit the large intestine. They are often effectually got rid of by rectal injections.

*Peritonitis.*—The peritoneum, like the pleura and the pericardium, is the subject of inflammations in which there may be much lymph coagulating and matting the parts together on the one hand, or on the other in which there is an abundant effusion of serum. The causes of it are generally ulceration of the alimentary tract, or inflammation of the generative organs. Tubercle growing in the mesenteric lymphatic glands or cancer in the bowel, etc., are also common causes of it. But inflammation in other organs of the abdomen, or bacteria introduced through wounds, etc., may also cause it. This is a most serious and fatal disease when the whole of the peritoneal sac is acutely involved. The only hope of preventing a fatal result lies in opening the cavity and washing it out. Hence when cases in which peritonitis is to be feared are under observation, the first and earliest indications of the supervention of peritonitis are watched for in order that the operation may be performed before the disease has got too firm a hold. Pain and collapse are sometimes signs of its onset. In tuberculous peritonitis very good results are sometimes got from opening the cavity.

*Ascites.*—Where there is obstruction to the flow of blood through the portal vein, from cirrhosis or other disease of the liver, or from lung and heart disease, fluid may be effused in large quantity into the abdominal cavity.

The usual means of relieving the patient of this fluid which presses upon his internal organs and causes great inconvenience is by tapping or paracentesis abdominis. This may be done by a small trochar and canula, or by Southey's canula which is left in for twelve hours or more, and gradually drains the fluid away. When the trochar is used, there is danger of the patient fainting from the sudden withdrawal of the fluid, and he must be kept recumbent. In using Southey's tubes, they need very careful cleaning, as they become blocked with coagulated lymph, every shred of which should be washed out of the several holes of the canula as soon as it has been withdrawn, and before it has had time to dry and harden.

*Jaundice* is due to disease of the liver or to obstruction of its duct. The skin is stained yellow, and if the tint be deep the skin may be affected with very troublesome itching. The urine is stained, and consequently the patient's garments become stained too. When the presence of jaundice is suspected, bile may be detected in the urine by testing it chemically before the bile pigment can be seen with the naked eye.

*Gall-stones.*—The bile deposits solids sometimes in the gall-bladder which form into stones, and these may be the cause of much trouble. If one escapes into the common bile duct, the flow of bile is obstructed and this causes jaundice. The effort made by the muscular coat of the duct to pass the stone onwards gives rise to paroxysmal pain and vomiting. The pain may be sufficient to double up the patient and throw him into a cold sweat. The gall-stones in the gall-bladder may cause inflammation and the formation of an abscess in the gall-bladder. The trouble thus caused can be relieved by an operation by which the gall-bladder is opened, and a stone lodged in the duct may be removed in the same way. If an operation be not performed, sedatives applied locally and internally are necessary to relieve the pain.

The gall-duct may be obstructed by inflammation, which is sufficient to cause this by the swelling of the mucous membrane which lines the duct, for this is very small. This is known as catarrhal jaundice and soon passes off. A permanent jaundice is caused by cancer obstructing the flow of bile. The malignant growth is nearly always secondary to cancer in the stomach or large bowel.

The substance of the liver may be the seat of an abscess, but this is rarely met with in this country. It is frequently seen in the tropics.

Cirrhosis is a common condition. It is due to the formation of fibrous tissue in the substance of the liver, which, shrinking as the fibrous tissue formed in the lung shrinks, causes wasting of the liver cells and puckering of the surface of the liver. Cirrhosis may cause hæmatemesis and eventually it gives rise to ascites. Chronic suppuration gives rise to the deposit of a substance in the liver, which may become very large in consequence. This is known as lardaceous disease. Cancer or hydatids may cause an enlargement of the liver also.

### The Nurses of the Royal United Hospital, Bath.

### A CHAT WITH THE MATRON. BY OUR COMMISSIONER.

ALTHOUGH the Royal United Hospital at Bath is a fairly large building, it is not very easy for a stranger in the city to discover it, because it is tucked away from the main thoroughfare and has no courtyard or gardens round it to attract the attention of the passers-by. The laundry and the chapel which have been added the most recently have taken up the last available space, and now, as the hospital is bounded on either side by a street, it is impossible for it to extend its sphere of usefulness except by removal. Like all the ancient houses in Bath there are quaint little rooms to be met with unexpectedly in odd places, and the matron's sitting-room into which I was shown had one corner occupied by a little passage built into the room in which was placed the entrance door.

Here Miss Polden, the matron, joined me, and I asked her if she could tell me why her hospital in particular was called "United."

"Partly," she replied, "because in it two useful charities, the Bath City Dispensary and Infirmary and the Casualty Hospital, were amalgamated in 1826, and partly, I should fancy, because it unites together so large an area, being the only large hospital for a good many miles round."

"How many beds have you, then?"

"One hundred and forty in all, eighteen cots being in the children's ward, which was added in 1890. This does not include the isolation wards, which are situated in an old house on the other side of the street."

"How long have you been matron of the hospital?"

"Since 1900. I was trained at 'Bart's,' and afterwards became a staff nurse there. Later I was night sister and assistant matron at the Poplar and Stepney Sick Asylum, with 770 beds."

"What is the extent of your nursing staff?"

"Twenty-eight in all; 21 nurses and six sisters, besides one theatre sister and an assistant matron."

### PERIOD OF TRAINING.

"The training is for three or four years?"

"For three years with a premium of £20, or for four years without premium. The fourth year, at the discretion of the Board, will be spent either in the hospital as a staff nurse, or on the private nursing staff of the Institution. When I came here the training was for two years in the hospital itself, the third year the nurse joining the private staff. But I did not think it was a wise plan. I find that it is during the third year in particular that I am able to judge what sort of nurse a probationer is likely to make. This is in great measure, of course, because by that time the novitiate stage is well passed, but also because opportunities are then given during the holidays for third-year probationers to take higher duty, and thus I am enabled to judge of their power of directing others and of acting on their own responsibility. At the end of the three years the nurses are given a certificate of competency, provided that they have passed their examinations creditably, discharged their duties satisfactorily, and generally conducted themselves well. Their certificate may be further endorsed at the end of the fourth year."

"Do most of the nurses leave you after their period of training is over?"

"Some of them seek a change, and others join our private nursing institution. Those who do so have two privileges, which I find are much appreciated. When there is pressure at the hospital, and an extra nurse is required, or for holiday work, they are given the opportunity of taking a special case, and thus they are able to keep their knowledge from going rusty and increase their experience by seeing the latest methods and the most up-to-date operations. Also when a vacancy occurs on the hospital staff they are eligible for election if they wish."

### SISTERS AND PROBATIONERS.

"You like to train your own sisters?"

"The position of day sister is filled from our own staff, and the winner of the Handley Gold Medal is invariably given the first chance. These medals, one gold and one silver, are bestowed each year on the two nurses who have most distinguished themselves during their three years' training. In making the awards the result of the examination by the medical and surgical staff and the general conduct and efficiency of the nurse during training, as shown by the reports of the matron, are both taken into account. The maximum marks are 300, of which 100 are given for medical nursing, 100 for surgical nursing, and 100 for general conduct. A probationer must gain 60 per cent. of the marks to pass, but no medal is awarded unless the nurse obtains 75 per cent. etc."

"Have you any difficulty in obtaining probationers?"

### THE NURSES OF THE BATH UNITED HOSPITAL, BATH—*Continued.*

"None whatever, and it is so difficult to reserve vacancies that I seldom promise to do so, filling up the positions as occasion arises."

"And the age limit?"

"Twenty-three to thirty. Each candidate has to serve for three months before her appointment is confirmed by the Board."

"To what class do your probationers mostly belong?"

"They are generally the daughters of clergymen, architects, and professional men, and it is a *sine quâ non* that they shall have been well-educated. A probationer finds that in listening to lectures, there is usually quite sufficient difficulty at first in understanding and grasping details and mastering long words and definitions without also having to struggle with an inability to spell correctly or to express herself clearly."

### Lectures and Salaries.

"How about the lectures to the probationers?"

"During the first year the subjects treated are practical nursing and physiology. I myself hold the classes, and the assistant matron instructs in bandaging, poultice-mixing, splint-padding, bed-making, etc. The resident house physician and the house surgeon also give lectures to second and third year nurses. In the second year more advanced lectures are given on the same subjects and the third year medicine and surgery are taught by the Honoraries, and massage classes are tried by a lady Masseuse. Every probationer has two months at a time in the theatre with the theatre sister, so as to gain practical knowledge as to her duties at operations."

"How soon do probationers take night duty?"

"Not at all if possible during their first year. They begin the second year and then they continue for three months at a time."

"What salaries do you pay?"

"None for the first year, and the probationers also pay for their own uniform. The second year they receive £8 and uniform, the third £10. This money is paid in monthly instalments, but it is understood that it is only made for a complete term of efficient service and satisfactory conduct. Anyone failing to give satisfaction to the medical or surgical officers or the matron will not be entitled to it. During the fourth year the salary is not less than £15, which may be increased at the discretion of the Board. As to uniform, it is easy to see at a glance how long a probationer has been in the hospital by it. The first year she wears pink and white galatea; the second blue and white; and the same with the addition of strings to the Dora cap, during the third year."

### Paying Probationers.

"Have you any paying probationers?"

"We occasionally receive probationers to train for private institutions, £12 10s. for one year, £25 for two years, and £30 for three years. They are given material for indoor uniform, but have to provide their own cloaks and bonnets. They must not be less than 23, and are in every way treated as the hospital probationers. At the end of their time, after examination, they are given a form to prove that they have spent a certain time at the hospital, with remarks on their conduct and the way in which they have done their work."

"What off duty time do you give?"

"Probationers have two afternoons a week, one evening from 6 to 8.45, in the third year to 9.45. Sundays 10 to 1 or 2 to 5, or 5.30 to 8.45. A half day every fortnight, a long day once a month, and 15 days the first year, three weeks the second year, the same the third year, and sisters one calendar month."

"Where do the nurses sleep?"

"At the top of the house. We have, unfortunately, no space to give each a separate bedroom, but each nurse has her own small cubicle. There is a sick nurses' room, and if a nurse shows any sign of being unwell she is removed there at once, as we find it better to let no one who is not perfectly well occupy a cubicle. The surgical or medical sister takes charge of the case. The nurses' sitting-room on the ground floor is provided with a bagatelle board and a piano."

### The Nursing Institution.

"There is a Nursing Institution in connection with the hospital?"

"Yes, and under my supervision. At present only two of our private nurses have been trained with us. The system introduced in 1900 of keeping our probationers here three years of course caused the private staff to suffer; but it was inevitable, as we could not get good work for the hospital any other way, and that was of course the first consideration."

"You tell me that nurses who join the Institution receive £30 per annum at first. Do their salaries increase?"

"Yes, by yearly increments of £2 10s. to £40. They also receive commission on their earnings—5 per cent. the first year, 7½ per cent. the second, and 10 per cent. the third and after."

### The Children's Ward and Chapel.

Before I left, the matron courteously showed me round the hospital. We went first to the children's ward, where nearly all the cots are endowed. I noticed that many of the Bath churches had a cot which they supported by their contributions (£20 a year), and that others had been endowed by friends in memory of a lost child. The children's sister had a little sitting-room for herself at the end of the ward, which, though not large was cosy and inviting. I remarked on the white counterpanes which I observed everywhere and was informed that all the linen throughout the hospital is supplied and made by the Royal United Hospital Ladies' Working Association, of which useful and admirable charity the President's able wife is the chief organiser. Last year this Association collected the generous sum of £75 towards the endowment of three cots in the children's ward."

As we passed to other wards, the small ward for cases of delirium tremens with double doors was pointed out to me, and lastly we came to the little Chapel. Here every morning at 8.30 the matron reads prayers to the nursing and domestic staff. Here also there is service on Sunday at 10, celebration at 8 A.M. on the second and fourth Sundays, and special Lent and Advent preachers arranged for by the kindness of the hospital Chaplain, the Bath Abbey Rector. A Bath lady plays the organ, and as I came out I remarked that two of the windows were filled with stained-glass. These I learnt were both tributes to Bath Hospital nurses. One had been put in by the Nursing Staff, the other had been contributed by the committee and the Nursing Staff in memory of the sister of the children's ward, who died last year.

### Wants and Workers.

Will any kind nurse give old clothing or uniform to a nurse in deep need, to enable her to return to her nursing again. Any help will be thankfully received by Nurse Ida, c/o 9 Gastigny Place, St. Luke's, E.C.

### Nursing Among Kentish Hoppers.

Last summer I was bidding farewell to hospital, having just completed my three years' training, when it was casually suggested to me that I might like to take up the management of a little temporary hospital for "hoppers." I knew nothing about hoppers, I had never even seen a hop garden, but the uniqueness, the independence of the project struck me, and the desire for varied experiences made me jump at the proposal. Having secured the promise of a friend to help me in such a nursing enterprise, I offered my services, and they were accepted.

I came home. I was asked on all sides, "Why do hoppers want nursing?" And as other nurses, as well as the general public, may like to hear the reply to this question, I will try and answer it, and, at the same time, give a brief account of our own experiences, with a description of our little hospital, and of the pickers at their work.

### Why do Hoppers Want Nursing?

Now to answer the question—Why do hoppers want nursing? Well, in the first place, it is not only the strong and healthy, but also the weak and sickly who go hopping—the consumptive father, the run-down mother, the anæmic daughter, and the ill-fed baby—and such, though benefited by the fresh air and sunshine, are not in a condition to stand cold and chill. Last year a terrible storm swept over Kent, the hoppers' tents got soaked through, there was no chance of a fire with the ground and wood all wet, and their garments (most of them had no change) took many days to dry. It was no wonder that coughs and colds, bronchitis, diarrhœa and sickness were very common, and that babies with croup, bronco-pneumonia, and numerous minor ills were brought up to the hospital.

### A Sad Case.

We had one very sad case of a little boy of two years old. I happened to be sitting up with a patient when, about 3.30 a.m., hearing a voice outside, I went to the gate and found a man who, in a voice choked with tears, begged me to come to his dying child. I called to my friend to look after the ward and started off at once with the father. We had more than two miles to go, and when we arrived at the tent the baby was lying stiff and unconscious after a fit of convulsions. The mother had thought of boiling some water, so I soon got him into a mustard bath (a pail doing service for a more elegant receptacle); this revived him to a certain extent, his muscles relaxed and the pulse improved, but his breathing was very rapid and shallow, and he did not look like lasting long. I felt I could not let the child die without the doctor seeing him; his house was only a mile off, so I wrapped the baby warmly up, and the father and I by turns carried our little burden there. But the poor little fellow was past all help; we took him back to the hospital and did our utmost for him, but in vain; he died in convulsions that afternoon.

### The Patients.

We were only called up at night on two other occasions, though during the day we were constantly fetched to go and see some one or other in the tents. We had six in-patients. The accommodation of our little ward was limited, and it was only those who were very ill that we could keep. One or two of our patients who either by accidents or owing to the effect of the climate, became unfit for work (this was the case with two anæmic girls), we managed to get sent back to their homes. It was among the out-patients, however, that our chief work lay. They came up at all hours, but chiefly between six and nine o'clock at night. During this time we were often both hard at work, bathing and binding up; and how funny some of those out-patients were!

### The Hospital.

And now for a brief description of the "Little Hoppers' Hospital." The cottage chosen for the purpose had formerly been a coachman's house, with coach-house and stables adjoining. The front room, with kitchen stove, table, and easy chairs, we soon made cosy and home-like; this, with two bedrooms above, comprised our private apartments. A door, always open, led through into the coach-house, which was our surgery and out-patients' department. Here was a large table holding lotions, drugs, washing-basin, etc., and with drawers where we kept lint, bandages, and other necessaries. There was a copper—this was a great boon, as it kept us supplied with hot water—and with rows of chairs for our patients, we had all that was required.

A ladder staircase brought us up to the ward. This mode of ascent at first sight struck one with dismay, but as we were only supposed to "take in" children, it did not really matter. The walls were whitewashed inside, the boards scrubbed clean; there was a stove, two beds, two cots, and a cradle, whilst a table and chairs completed the furniture of this modest little room. The ventilation was good, and some bright hangings and a curtain across the door gave it a cheery appearance.

### The Foreigners.

The hop season was late in 1902. It was not until the second week in September that the "foreigners" as the exclusive Kentish folk call the strangers who come from London and elsewhere for hop-picking, arrived, and we opened our hospital. The hop-pickers come by road and rail, some a-foot, others in their barrows, traps, or house carts. Whole families come down; it is their summer outing; they get fresh air with a change of work, which will pay expenses and should leave a little over. Arrived at their destination they seek the tents or sheds which are provided for them. These are only places for sleeping in; they cover the ground with faggots and over this spread a thick bedding of straw. All cooking is done outside, or in sheds provided for the purpose. The people go to work at six o'clock and usually are "called off" at the same hour at night.

If the weather is bright and warm life goes merrily in the beautiful hop gardens. All pick busily—they are paid by the quantity they pick—into their bins; even the babies help, if not by picking, at least by adding their shrill notes to the strains of "Dolly Gray" and the "Honey and the Bee." The hoppers are charmed if you talk and pick with them, especially our Borough friends; the home pickers are inclined to be superior. The children with their little worn, precocious faces of the cockney type, such a contrast to the healthy country children, much impressed me. All paid due respect to our uniform, and we felt that we were always welcome.

Our chief adviser was the rector, to him we went for everything, and always got what we wanted. The doctor, too, kindly gave his services; he looked into the hospital most days and always came when we sent for him. I must not forget to mention our cook whose assistance was also voluntary, and whose help in many ways was most valuable.

The Hop-picking Committee paid all our expenses. I believe they had twelve nurses working in different parts of Kent, besides other workers; ours was only a part of the work, but a very necessary part, and I think that any nurse who can spare the time would thoroughly enjoy the experience and the feeling that she is of some little use to a class of people with whom she might not otherwise come in contact.

### Everybody's Opinion.

[Correspondence on all subjects is invited, but we cannot in any way be responsible for the opinions expressed by our correspondents. No communication can be entertained if the name and address of the correspondent are not given as a guarantee of good faith, but not necessarily for publication. All correspondents should write on one side of the paper only.]

### LINSEED POULTICES.

"Sister" writes: In your issue of July 11th I read the answers of both nurses to the question of how to make and apply a linseed poultice. As a nurse of nearly twelve years' experience, I consider that it is a very old-fashioned way of covering a poultice with jaconnette. In the first place, it is altogether unnecessary for keeping the heat of the poultice, as cotton wadding is quite sufficient for that purpose; and, secondly, it has a very bad effect on the patient's skin, making it very sodden and quite unfit for any further applications.

### ANTISEPTIC HAIR-DRESSING.

"M. R." writes: Referring to your recent article on antiseptic hair-dressing, I wonder how most nurses disinfect their hair when they go out while nursing any infectious case in hospital or private house? The caps worn by most nurses do not in the least protect the hair. During the time I was at one of the Metropolitan Asylums Board hospitals I could not help wondering why they did not arrange to keep the nurses who were attending different diseases apart? We only had one room for meals and recreation and rest. I am sure it was running an unnecessary risk to nurses and their patients that nurses from enteric, scarlet fever, and diphtheria wards should all mess together at meal time and then return back to the various wards. Then, again, it was a very common thing to see nurses come into the mess-rooms to supper in their outdoor dress, thus coming in very close contact with the nurses straight from the various infection wards. Sometimes, too, without any previous warning, a nurse went to the office to report for duty, and without any warning or preparation she would be sent to another sphere of duty—maybe to an enteric ward—in the same clothes as she had worn for the previous three or four days in a scarlet fever ward. We had no kind of cupboard or wardrobe provided for our clothes, and it was quite a common thing to see the nurses' uniform dresses and their outdoor garments hanging together on the door of their room. Of course things are better managed now. But I could not help being struck at the time with such utter disregard of danger to others.

### PRIVATE NURSES AND WINE.

"District Nurse" writes: When reading your note "Private Nurses and Wine" I could not help wondering why that nurse chose nursing for her vocation, though it must be remembered that nurses' of some years standing know perfectly well that even "threadbare carpets" and other tokens of poverty are sometimes signs of extreme meanness on the part of the owners who will feed on the best of food and drink the best of wine, while the nurse is offered nothing but the plainest of fare. I am speaking from experience and I know "that things are not always what they seem." Then again, I do not think that it is right for any institutions to state "that neither wines nor spirits shall be given to the nurses except by medical directions." If a nurse is fit to be entrusted with the care of an extremely ill and helpless patient, she is quite fit to take wine with her lunch or dinner. I can imagine nothing more embarrassing for a woman, say of 30 or 40, who from her girlhood and still at her own table did and does take a glass of wine with her lunch and dinner, sitting at table with her patients' relatives or friends who are taking wine or champagne and the nurse is to drink cold water unless they ask the doctor. Candidly I should break the rule. But I would not be stupid enough to sign it because I consider it is an insult to self-respecting women, who should be able to be trusted to know when and what to accept or refuse. Nurses are often placed in very awkward positions. There is surely no need to send them to cases branded as women from whom wine or spirits must be withheld. In all my experiences I have met a good many nurses, and never yet one who drank to excess.

### A NURSE'S REPUTATION.

"A Nurse in South Africa" writes: It has struck me lately that our reputation as nurses is very easily lost and won. We share the precarious opinion of good or ill with medical men. A true diagnosis of a hitherto baffling ailment, a diagnosis made, more than likely, by one of those sudden sparks of intuition, which suddenly illuminate the darkness, will often make a doctor famous, and a lucky hit in the treatment of a disease lifts him at once to a pinnacle of fame and brings patients by the score to test his marvellous powers of discrimination. A nurse may render services in the ordinary way, but with such happy results that immediately her name is made, and if her patient has a large circle of influential friends the nurse's connection is secured, and she is lauded to the skies. Take the other side, which is the same with doctor or nurse. A skilful, conscientious physician attends a case; it is a serious one, and he puts forth all his powers of mind and learning to fight the grim phantom which he knows is near. He comes three or four times a day to the house of his patient and carefully notes each symptom, regulating the treatment accordingly. He is baffled!—for nature will cheat even the very wisest of men, and the anxiety and doubt follows him as he goes his rounds, and all day long his mind reverts to that pain-racked form. His brow grows furrowed into lines of anxious care, and his voice is less cheery and his smile less bright. The end comes, and alas! his skill is unavailing, and his reputation in that quarter is gone. "Oh, if only we had called in Dr. R. our dear one would have lived. I don't believe in Dr. M." Poor Dr. M.! it would be little wonder if you grew hard and bitter when this is all you can expect in return for hours of anxious thought and care. And so it is with the nurse; she may be most faithful in the discharge of her duties, she may conform most rigidly to all the doctor's rules and regulations, and follow, to the letter, all his instructions. She may have weary hours of strain, and never once relax in her efforts to ease and comfort, and never change her watchful attitude. It is her part to bear the responsibility, through the most critical period of the disease. And yet, in many cases, an unsatisfactory symptom, a sudden rise of temperature, an unlooked-for complication, will often be laid to her charge. "I don't trust Nurse L., she seems all right when we are at hand, but how can we tell what goes on when she is left alone? My poor child is so much worse this morning, and doctor cannot account for it." Nurse L. is not trusted, and her patient care and watchfulness is not noticed or appreciated. No one must think, when reading what I have written, that I believe one's efforts are never appreciated. I thank God there are many people who see far enough to recognise true faithfulness and merit, both in doctor and nurse; but there are others who do an infinite amount of harm through speaking unadvisedly—who, without in any way knowing the circumstances, give an opinion for or against a doctor or nurse! Some time ago I was very much surprised and grieved when a lady said to me, "I hear Nurse S. is a terrible person; they were discussing her at Mrs. A.'s yesterday. She almost killed poor Mrs. B." As it happened, Nurse S. was a friend of mine, and I had always heard her most highly spoken of. I therefore made it my business to go and see the doctor who had attended Mrs. B., and asked him to be kind enough to tell me the true facts. I am glad to say he entirely exonerated Nurse S., and said such a statement as that made by Mrs. B. was quite untrue. I tried to circulate the truth about my friend, but already the story told in Mrs. A.'s drawing-room had done her serious injury. It is hard to think that the wagging of careless, I will not say malicious, tongues has power to seriously affect the career of doctor or nurse. So far I have been wonderfully fortunate; but I quite expect to have my turn of unpopularity and disfavour. One must live as it were on the brink of a professional precipice, and an unthinking word or a thoughtlessly given opinion may push us over and cause us to sink into obscurity, or worse than that, into disrepute. The tongue is a little member, but how great a matter a little fire kindleth.

### Appointments.

BIRMINGHAM HOMŒOPATHIC HOSPITAL.—Miss Annie A. Mossop has been appointed lady superintendent. She was trained at the General Hospital, Birmingham, and has since been sister of the male surgical ward and sister of a female ward, home sister, and night superintendent. During holidays she has acted as housekeeper.

BROMLEY HOMŒOPATHIC HOSPITAL. — Miss Helen F. Campion has been appointed staff nurse. She was trained at the Mater Misericordiæ Hospital, Dublin, and has since been attached to St. Mary's Nursing Institution, Shrewsbury.

CHALMERS HOSPITAL, EDINBURGH.—Miss Priscilla Louisa Lawrence has been appointed sister. She was trained at the Royal Hospital, Sheffield. She has since been holiday sister at the Royal Hospital, Sheffield, and has been attached to a West End Nursing Home in London.

DARTFORD FEVER HOSPITAL.—Miss Margaret McLellan has been appointed matron. She was trained at Perth Royal Infirmary, and has since held the posts of charge nurse, night superintendent, and housekeeper at the Brook Hospital, Shooter's Hill.

ENFIELD COTTAGE HOSPITAL.—Miss S. M. Messum has been appointed nurse matron. She was trained at Sunderland Infirmary, and has since had charge of the women's wards at the General Hospital, Tunbridge Wells, and been nurse at the Royal Berks Hospital, Reading.

HACKNEY UNION INFIRMARY.—Miss Louisa Mary Stoward has been appointed assistant matron. She was trained at the General Infirmary, Leeds, where she was afterwards nurse. She has since been sister at the Yorkshire Co-operation for Nurses, and sister at the Cambridge Hospital, Aldershot.

HOSPITAL FOR INCURABLES, NEWCASTLE-ON-TYNE.—Miss Rosa D. Smith has been appointed charge nurse. She was trained at the Leeds Union Infirmary, and has since been for a short time charge nurse at the Newcastle-on-Tyne Union Infirmary. She has also done private nursing.

PERSHORE ISOLATION HOSPITAL.—Miss Mary J. Hopton has been appointed nursing superintendent. She was assistant nurse at the Poplar and Stepney Sick Asylum and was afterwards trained at St. Marylebone Infirmary, London. She has since been staff nurse at the Central London Sick Asylum, Cleveland Street, W., private nurse at Brighton, on the staff of the Nurses' Co-operation for eleven years, and nurse in charge at the Pershore Temporary Hospital.

ROYAL VICTORIA HOSPITAL, BELFAST.—Miss Alice Hilson has been appointed assistant matron and home sister. She was trained at the Western Infirmary, Glasgow, and has since been ward sister at the Chalmers Hospital, Edinburgh, and matron of the George Square Nursing Home for Women, Edinburgh. For more than three years she has been employed on the Army Nursing Service Reserve, both in South Africa and the Curragh.

SUNDERLAND UNION WORKHOUSE INFIRMARY. — Miss Jane Agnes Ellithorne has been appointed charge nurse. She was trained at the Sunderland Infirmary, and holds the L.O S. certificate.

### Presentations.

CUMBERLAND INFIRMARY, CARLISLE. — Miss Hodges, matron of the Cumberland Infirmary, Carlisle, on leaving to take up her duties as matron of the Royal Infirmary, Bradford, has been presented with a very handsome gold chain by the nursing staff as a token of their esteem and good wishes.

### The Nurses' Bookshelf.

MEDICAL TERMINOLOGY: HADDEN'S POCKET VOCABULARY OF MEDICAL TERMS WITH THEIR PRONUNCIATION. By Henry Payne, M.D., M.R.C.S., L.S.A. Revised and enlarged by Philip Owen, M.D. (Durh.), D.P.H. (Camb.) and Herbert Davey, of the Middle Temple, Barrister-at-law. Third Edition. (London: Hadden, Best and Co. 1902.)

OF the making of medical dictionaries there is no end—good, bad and indifferent blossom forth in rich abundance. But this modest little "vocabulary" has already lived to reach "years of discretion." The fact that since its first appearance it has twice undergone revision, and is now in its third edition, testifies that it has met "a distinct want." Its original object was to provide for registrars, poor-law officials, and others, who were not members of the medical profession, but who in the course of their duties were brought into contact with medical terms, a concise and convenient pronouncing dictionary. In the present edition the work has been revised and extended. There is a useful introduction indicating the formation of many words in universal use. The list of Greek and Latin roots, prefixes, and terminals used in medical terms should prove of much value. The vocabulary is judicious and likely to be useful. An alphabetical list of diseases which may result in death will afford considerable help to registrars. The work in spite of a few slips is praiseworthy, and in addition to those for whom it is particularly intended, nurses, students, and ordinary laymen may find much that is likely to be of interest and service.

PRISON HOSPITAL NURSING. By HERBERT SMALLEY, M.D. Published by authority. (Pp. 344. 51 illustrations).

THIS well-written and interesting volume on prison nursing comes opportunely to those who read (with more or less agreement) an article published in our pages on April 25th on the same subject, headed "The Nurse in Prison." In both cases the authors speak only of the nursing necessary to their own sex, which is natural, for if we understand the question rightly, at present no female nurses are allowed in the prisons for men. Both writers appear to look upon prison nursing as if it differed greatly from the same work with other classes. Surely this is an error. Dr. Smalley's book is excellent, and as a manual for male nurses it seems nearly perfect—the illustrations are admirable—the text simple, comprehensive, and so well expressed that the dullest student must find it easy to understand the instructions. Of the making of nursing manuals there is no end, and though the excuse for this brilliant little book is the benefit to be derived (through its perusal) by the prison nurse, after all, the adjustment of splints, application of bandages, poultices, etc. . . . and the arrest of hæmorrhage must always be the same whether the patient be sinner or saint. The figures illustrating the position of arteries are very good, and so are those on bandaging—most clear and descriptive; but we fear no human hands will ever succeed in applying the "simple spiral" so picturesquely to an arm unless it resembles a broom handle in shape! The chapters in Part II. on "Crime and Criminals" and the "Classification of Criminals" are most interesting even to outsiders, and show an honest desire to do the very best for these unfortunates, without lapsing into the somewhat sentimental attitude of the writer of the article in the columns of THE HOSPITAL, who appears to place the nursing of criminals above all other similar work in different classes. Dr. Smalley's book will be found to be useful; he sweeps well into the recesses of our minds, and leaves no unilluminated corners.

### Echoes from the Outside World.

### The King and Queen in Dublin.

On Wednesday, last week, the King and Queen held a State reception in St. Patrick's Hall at Dublin Castle, when deputations were introduced and addresses presented from the citizens' reception committee and many other public bodies. At the conclusion of the ceremony the Queen left the Castle and visited the Alexandra College, where she presented certificates to distinguished students. On Thursday morning the King presented colours at the Viceregal Lodge to the Royal Hibernian Military School and addressed the boys. At noon he reviewed the troops in Phœnix Park. On Friday morning the King visited the Guinness Trust buildings in Patrick Street, St. Patrick's Cathedral, and the Corporation Buildings. Later in the day the King and Queen proceeded to the Royal Irish Constabulary depot, visiting in the afternoon the Royal College of St. Patrick, Maynooth. On Saturday their Majesties left Dublin, driving in an open carriage from the Viceregal Lodge to Amiens Street Station. Although the weather was unfavourable, the streets were lined with spectators, and their Majesties were received all along the route with enthusiastic cheering. Mr. George Wyndham, Chief Secretary for Ireland, has addressed by the King's command a letter to the Lord-Lieutenant expressing his Majesty's appreciation of the loyalty and affection with which he and the Queen have been surrounded during their stay in the Irish capital.

### The King and Queen in Ulster.

On Saturday the King and Queen arrived at Mount Stewart, the seat of Lord and Lady Londonderry. On Monday they visited Belfast, and were welcomed with great enthusiasm by immense crowds of people. At the station an address was presented from the Lord Mayor and Corporation, which the King acknowledged, expressing the hope that his peoples, "cherishing their own national characteristics and ideals, may each engage in that friendly rivalry in the paths of peace which is the true source of national prosperity and Imperial greatness." Their Majesties then drove to the front of the new Town Hall, where the King unveiled a statue of Queen Victoria. The proceedings of the day included a luncheon at the Town Hall, and a visit to the North-East Agricultural Show at Balmoral, where an address was presented. On Tuesday their Majesties proceeded by rail from Donegal to Londonderry, where more addresses were delivered. In the afternoon the King and Queen each planted a tree in commemoration of their visit.

### Incidents of the Royal Visit to Ireland.

On Friday when the King paid a visit to the poor quarter of Dublin, in the neighbourhood of St. Patrick's Cathedral he wore a green tie with a shamrock pin. Having inspected the Iveagh dwellings his Majesty passed out into the street, and one of the Royal servants grasped the handle of the carriage door, but the King shook his head. "No, I am going to walk," he said, and walked down the narrow little street, followed by the Chief Secretary and the Lord Lieutenant, the people crowding round. On leaving the Cathedral, after inspecting the monuments of Swift and Stella, an old woman stepped forward. The King's attendants were pulling her back, but the King interposed, saying, "Leave her alone, God bless her," and shook her heartily by the hand. The King paid a visit of some length to the artisan's dwellings, and chatted with several of the inmates. He first went to a little tenement of three rooms, where Mary O'Neill and her three children lived. He shook hands with the mother and said he was pleased to see her, adding, "And these are your children! And fine and healthy they look, too," giving the eldest girl of 10 a playful tap on her cheek. On the wall of the dwelling-room the King saw his own portrait, and in the bedroom was one of the late Pope. The King said that he was pleased to see the portrait, and added, "The Pope received me very well when in Rome, and I shall not soon forget it." The King has made a gift of £1,000 to the poor of Dublin.

When the King and Queen left Dublin on Saturday, on their way to Newtownwards to visit the Marquis and Marchioness of Londonderry, they drove in an open carriage, notwithstanding that a drizzling rain was falling. After leaving the Viceregal Lodge, 18,000 children had gathered in the park, the majority having come from the poorest parts of the city. A bouquet was presented by two children who had been selected haphazard as representing the classes for whom the fête was mainly intended. One was Henry Box, a Roman Catholic, and the other Florrie Martin, a Protestant. The little ones appeared barefooted and in the tattered garments which were their everyday attire. They were too small to lift the bouquet into the carriage themselves, and a gentleman usher had to assist them.

In the course of the drive to the Victoria Hospital, Belfast, on Monday afternoon, an old woman attempted to cross the road in front of the Royal carriage. She was knocked down by one of the horses of the escort, and severely hurt. The King at once had the carriage stopped, and sent a footman to inquire as to the woman's injuries. Later he telegraphed to the hospital for further information.

### Burial of the Pope.

The temporary interment of the body of Pope Leo XIII. took place on Saturday night in the Basilica of St. Peter's in presence of the College of Cardinals and about a thousand privileged persons. The funeral ceremonies occupied over two hours. The body having been laid in the coffin there were also placed in it a parchment scroll, enclosed in a tin case, containing the name, age, and a brief life-sketch of his Holiness, and three silken bags, each containing 25 medals, respectively gold, silver, and bronze, the number of years during which he governed the Church. When the cypress-wood coffin had been screwed down, it was enclosed in two others, one of lead and the other of elm. On the leaden case seals were affixed with the coat of arms of the Camerlengo, of the Majordomo, and of the Vatican Chapter, three seals on the right hand and three on the left. On the outside coffin is represented a cross with the tiara, but without the keys, and with the private seal of the deceased Pope, the only one which is never broken by the Camerlengo.

### Railway Accident in Glasgow.

A remarkable and disastrous railway accident occurred at Glasgow on Monday morning. As a heavily-laden express train, conveying excursionists returning from the Isle of Man, entered St. Enoch's station, the terminus of the Glasgow and South-Western Railway, at a high rate of speed, it dashed against the buffers with such force that the two coaches next to the engine were telescoped, the second being completely destroyed. A heartrending spectacle was formed by the confused mass of killed and injured passengers, heavy luggage, and débris, and the moans and cries of the injured were sickening. Medical men, railway officials, and passengers by other trains joined in the work of rescue; and a waiting-room was turned into a mortuary. Several of the injured had to be literally cut out of the wreckage. Fifteen persons were killed, including an entire family of four; and twenty-five more or less injured. The driver of the train, who blames the brakes for failing to act, is in custody.

### A Book and its Story.

### AN ARTIST'S IMPRESSIONS OF THE HOLY LAND.*

Lady Butler's "Letters from the Holy Land," issued in one volume by Messrs. A. and C. Black, is a charming book. The 16 illustrations are very delicately reproduced from sketches made on the spot, and printing and binding are both alike admirable.

Brought face to face with holy places and hallowed scenes the most attractive book on the sacred country fails to hold the reader. It is Bible Land, and "In this ancient land which is yet so fresh—so fresh in its surprises and in its stirrings of the heart—no book of human authorship dealing with the subject is readable. You may take any of the delightful works that have fascinated you in times gone by; you will open them once but not again. *The Bible is the only Book you can read here!* All others are inadequate; no man can measure himself with the Infinite." At every turn some text in the Old or New Testament which alludes to the natural features of the land springs before one's mind illumined with a light it could not have before. I can best describe the fascinating quality of our journey by saying it is like riding through the Bible. I know many devout Christians shrink from a visit to the Holy Places for fear of—what? Do not fear! The reality simply intensifies, gives substance and colour to the ineffable poetry of the Bible. It is simply rapture to see the last originals of our childhood's imaginings, and, believe me, the reality becomes more precious in one's memory, even than the cherished illusion." The Lake of Galilee was visited on a soft spring day. "The lake was pale and calm, delicately tinted, and there was a heat-haze over everything in the early part of the day. We disembarked under some thorny acacias, which gave a deep shade, and I had the delight of making a sketch there of the coast looking westward. Rosy oleanders fringe the water as far as the eye can reach; the 'Mount of Beatitudes,' and the top of Tabor are in the distance, and the site of Capernaum in the middle distance. God has trodden these scenes with human feet; the feeling of sketching them is scarcely to be put before you in words."

Of springtime in the Holy Land Lady Butler, writing in the vicinity of the Plain of Sharon, says: "The soil of the country now being ploughed in all directions between the green wheatfields is of a rich golden colour, like that I noticed with such pleasure round Sienna, and makes a pleasing harmony with the vivid green. The dear olive-tree, beloved of my childhood, is here in its very home. I hailed its pinky-grey foliage and its hoary old gnarled trunk. And now for those wild flowers that all travellers who are so well advised as to come here in spring have told us of. Well may they speak of them with rapture! As we proceeded they increased in variety and so abundant were they that they made tracts and wide regions of colour over the land. Come here in spring, O traveller! and not in the arid, dusty, burnt up autumn."

On arriving at Ramleh a magnificent view was seen from the top of the great tower of the whole of Philistia. Northward lay Carmel, westward the sea, eastward the mountains of Judah. "As the sun sank the tints deepened on that lovely plain, and nothing on earth could be more beautiful than that immense view. As we walked I had become absorbed in the contemplation of the limpid sky where the last lark was carolling to the sinking sun, and of the mountains, whose rosy flush was fading into the cool greys that already veiled the plain, when my eyes sinking lower, I beheld in the cold grey of the narrow street, ranged along a stone wall, a row of lepers waiting for alms." Life has no sadder detail than the leper. The description that follows is too painful to quote. After receiving the money offered by the travellers, "A low wail passed along the line, and bony arms were stretched out in gratitude."

When looking towards Jerusalem from one of the commanding hills the view filled the mind of the spectator with thoughts of all the tragic scenes the Holy City had passed through. "If we could really concentrate our thoughts upon the events that have passed upon this ground, which, from such a standpoint as ours of this morning, the sight can compass in one sweep of vision, it would be too painful to be endured. Perhaps if I could see the place on some bleak twilight or in a sounding thunderstorm, I might dimly appreciate the long agony of Jerusalem, but to-day the April air was full of scents of flowers and aromatic shrubs, the bees were humming, and there were little butterflies among the anemones. The very spirit of gospel peace seemed to float in the gentle air of spring." Lady Butler encourages intending travellers with promises of finding in the Holy Land few of the disenchantments associated with the idea of the country as it is to-day. They will be more than rewarded for a few difficulties and fatigues by the simple reality of their surroundings.

Among things imported into Palestine and looking strangely out of place which caught Lady Butler's eye when being whirled along in a vetturino drawn by three horses abreast in company with other jingling whip-cracking vehicles, escorted by horsemen in brilliant Syrian costumes, carrying long ornamental guns slung across their backs, was a glimpse "just as we started, of a railway engine, under some palm trees. It was waiting there the completion of the line to Jerusalem to puff and whistle its beauty-marring career to the Holy City."

In referring again to Jerusalem in a later letter, and to the traditional sites of Calvary and the Holy Sepulchre, Lady Butler says: "I told you how convincing one feels the traditional sites to be in spite of modern scepticism. But more modern still is the verification of their authenticity. It has lately been proved by that 'research' which we seem to require nowadays that they stand *outside* the line of the city walls of our Lord's time. The great stumbling-block to those who could not accept the word of tradition consisted in the idea that those sites had always been within the city as now, for it is known the Jewish law forbade places of execution and of burial within the walls." The outline of Jerusalem, marked by its fortifications, has so changed since our Lord's time that "whereas the house of the Last Supper stood within the lines then, it stands far outside the walls of to-day."

Nearing the end of her journey, in the presence of an Eastern afterglow, so subtle, so powerful, and yet too low to allow the artist to see well enough to sketch in it, she records her final impressions. "Of all the splendours of nature the afterglow is the most surprising. . . . The sky becomes a low-toned grey-green blue, against which the illuminated objects on earth shine with the colours of flame rather than that of the sun." . . . Then the writer refers to the ringing of the Angelus up at the Carmelite Monastery, of which she says: "The lonely monastery with its monks chanting Latin to nobody only to the great God who chose their little country wherein to testify His love for men; who has trodden with weary feet those hills we have traversed in the journey that ends to-day!" The authoress brings the spirit of reverent inquiry to bear on every scene and place visited, and there is a quiet charm in the artistic record of her impressions, which cannot fail to delight the reader. She touches lightly on details that strike a discordant note, and debar many people from undertaking the journey to Palestine. She lingers only over things that are lovely, and while not ignoring the existence of drawbacks, allows them only a proper and subordinate place in the whole scene.

* "Letters from the Holy Land." By Elizabeth Butler. (A. and C. Black. Price 7s. 6d. net.)

### For Reading to the Sick.

"GOD WITH US."

Shut out from life's fair joys,
From spring's bright flowers,
From summer's smiling scenes,
With weakened powers;
I lie a prisoner in my little place,
And sing God's grace.

Shut out from life's sweet toil,
Shut in with pain,
Yet Thou hast changed each loss
To brightest gain;
And Thou hast taught me, though to learn so slow,
This truth to know:

Whether by fervent toil,
Or lying still,
He only truly serves
Who does Thy will:
Then where and how Thou wilt, Thy servant use;
I would not choose.

*S. M. G.*

Throughout His earthly life our Lord Jesus Christ was in a unique sense alone. In the days of His ministry neither His best friends nor His nearest disciples could understand Him; He was alone. He experienced as no other could, the isolation of human life. . . . Alone in spirit though often surrounded by a multitude, for, after all, there is no solitude like being alone in a crowd. He was alone in His greatness, for He was the Perfect Man who knew no sin; alone in His sorrow, for, "Behold and see if there be any sorrow like unto My sorrow," and alone in His death. Now, at whatever immeasurable distance, this feature of solitude finds its counterpart in every human life.

Isolation is meant to throw us back increasingly upon God. We have learnt to look beyond the transitory and the seen, to what is unseen and eternal.

Do not let us abandon that vision! What we see passes away, that endures which we cannot see. If the burden of isolation presses, remember that those whom you see around you are but shadows; but He is near you by His blessed Spirit Who endured for us all the isolation of the Passion, and Who, in the agony of that supreme isolation, "poured out His soul unto death."

The most fruitful moments may not be those of speech, but those of silence, of silent prayer and meditation. Make the most of these and expect God then to send a message into your soul. Say, "I will *hearken* what the Lord God will say concerning *me.*"—*Randolph.*

Fear thou not; for I am with thee.

Oh, say not thou art left of God,
Because His tokens in the sky
Thou can'st not read! This earth He trod
To teach thee He was ever nigh.
And when thou liest by slumber bound,
Outwearied in the Christian fight,
In glory, girt with saints around,
He stands above thee through the night!

*Newman.*

### Notes and Queries.

**FOR REGULATIONS SEE PAGE 219.**

*Home.*

(180) Can you tell me of any home where a girl of 17, a domestic servant, could be received? She is suffering from phthisis.—*Nurse F.*

The Mount Vernon Hospital for Consumption, Hampstead, N.W. Of course you must obtain a Governor's letter. For other special hospitals for consumption see "Burdett's Hospitals and Charities."

Can you kindly tell me of a good home where a young man, suffering from spinal caries, could be received as a permanency? 10s. a week could be paid.—*Verity.*

See list of homes for incurables in "Burdett's Hospitals and Charities." Perhaps the case is suitable for one of the homes under the direction of St. Peter's Home and Sisterhood, Mortimer Road, London, N.W.

*Maternity Fees.*

(181) Can you tell me what a monthly nurse would charge a lady? I have the L.O.S. certificate, and wish to obtain monthly nursing.—*M. R.*

Fees vary according to the circumstances of the patient. Usually from £4 4s. upwards.

*Legal Hours of Duty.*

(182) Would it be legal for a private nurse to do 18 hours continuous duty?—*Nurse B.*

Certainly it is legal; but it ought not often to be necessary.

*Birmingham.*

(183) Will you kindly inform me if there is a Nurses' Hostel in Birmingham?—*Inquirer.*

Not at present, though there has been some talk about founding one.

*America.*

(184) 1. Will you kindly tell me the names of some good asylums in America? 2. Are ladies employed as mental attendants, and (3) is the treatment of the insane more advanced in America than in England?—*G. C. V.*

1. You will find a list of some 150 American asylums in "Burdett's Hospitals and Charities." 2. There are ladies amongst the attendants. 3. In some respects.

*Hollond Institute.*

(185) I should esteem it a favour if you would let me know the address of the Hollond Institute.—*R. W.*

The Hollond Institute is now the Nice Nursing Institution, Villa Pilatte, Avenué Désambroir, Nice.

*South Africa.*

(186) Will you kindly give me the address of the Committee sending domestic servants to South Africa?—*M. D.*

The South African Expansion Emigration Committee, 47 Victoria Street, S.W.

*Midwifery.*

(187) Will it be necessary for one holding Queen Charlotte's Hospital certificate, and who has been four years district midwife, to take the L.O.S. in order to be registered?—*K. A.*

No.

*Sterilising a Syringe.*

(188) I shall be glad if you can tell me the easiest and quickest way to sterilise a new indiarubber enema syringe. It has patent valves which I am afraid I might injure if I boiled in the ordinary way. I have not the use of a steriliser.—*Sister Dora.*

If the valves are of metal it will not damage them if you boil the apparatus. Otherwise the best thing to do is to wash the syringe well with soap and water and then let it lie for 15 minutes in some 1-40 solution of carbolic acid, having first pumped some of the solution through the syringe. Care must be taken to thoroughly empty the syringe of disinfectant before it is used. The nozzle should always be boiled.

**Important Nursing Textbooks.**

"The Nursing Profession: How and where to Train." 2s. net; 2s. 4d. post free.

"A Handbook for Nurses." (New Edition). 5s. net; 5s. 4d. post free.

"The Human Body." 5s. post free.

"Ophthalmic Nursing." (New Edition). 3s. 6d. net; 3s. 10d. post free.

"Gynæcological Nursing." 1s. post free.

"Art of Feeding the Invalid." (Popular Edition). 1s. 6d. post free.

**"Practical Hints on District Nursing." 1s. post free.**

